# Alexithymia and Stress Response Patterns among Patients with Depressive Disorders in Korea

**DOI:** 10.4306/pi.2009.6.1.13

**Published:** 2009-03-31

**Authors:** Hea Won Kim, Hyo Deog Rim, Ju Hee Kim, Seung Jae Lee

**Affiliations:** Department of Psychiatry, School of Medicine, Kyungpook National University, Daegu, Korea.

**Keywords:** Alexithymia, Depression, Stress

## Abstract

**Objective:**

Alexithymic characteristics may represent cognitive and affective mediators between stressors and stress responses among those with depressive disorders. This study evaluated how alexithymic characteristics, as measured by the Korean version of the Toronto Alexithymia Scale-20 (TAS-20K), could be related to stress response patterns, as measured by the Stress Response Inventory (SRI), within a sample composed of individuals diagnosed with depressive disorders.

**Methods:**

Participants comprised a cross section of patients diagnosed with depressive disorders (n=98). Data on demographic and psychosocial factors (i.e., sex, age, and level of education), clinical profiles {i.e., primary and comorbid psychiatric conditions meeting the Diagnostic and Statistical Manual of Mental Disorders, fourth edition (DSM-IV) criteria at the time of the evaluation}, duration of illness, medications, and Clinical Global Impression (CGI) scores, and the results of psychological assessments (TAS-20K, SRI) were analyzed.

**Results:**

Patients having depressive disorders with alexithymia obtained significantly higher scores in terms of all seven subscales of the SRI, as compared to those without alexithymia, a logistic regression model was used to assess possible predictors for the presence of alexithymia in patients with depressive disorders, including the seven subscales of the SRI, gender, age, and duration of illness. We found that aggressive and somatizing responses to stress were significantly associated with the presence of alexithymia among patients with depression.

**Conclusion:**

These findings suggest that patients having depression with alexithymia were more susceptible to stress than those without alexithymia. Clinicians might improve their treatment of depression by identifying the clinical predictors for alexithymia and by helping those individuals demonstrating such symptoms in coping with emotionally stressful situations.

## Introduction

Despite the wide variations characterizing the use of the word, Lazarus^1^ emphasized four essential concepts to describe the stress process. These included an external or internal causal agent (stressor); an evaluation performed by mental or physiological systems distinguishing noxious from benign phenomena and events, coping processes by which the mind or body deals with stressful demands, and a complex pattern of mental and physiological reactions (stress response). Recently, explanations of individual differences in the stress response have placed more emphasis on cognitive appraisals, coping abilities, illness behaviors, and social supports than on recent life changes (stressors).^2^-^6^

Depression has emerged as the most likely psychiatric illness to fit the stress-diathesis model. Stressors were 2.5 times more likely to be reported among patients with depression than among controls, and the onset of depression in community samples was preceded by major life events in 80% of the cases.^7^ Significant associations between prior stressors and depression have also been confirmed.^8^ In addition, the stress response scores of patients diagnosed with depressive disorders were higher than those of other diagnostic groups, and the former also reported higher levels of tension and anxiety compared to patients with anxiety disorders.^9^ Severe or chronic stress has been suggested to cause an overall depression in intellectual functioning, resulting in cognitive distortions, misinterpretation of situations, unproductive and ineffective thought patterns, and indecisiveness, thus bridging the gap between stressful stimuli and stress responses among individuals with depressive disorders.^10^

Alexithymic characteristics might represent additional cognitive and affective variables that mediate between stressors and stress responses among patients with depression. The term "alexithymia," literally meaning "no words for mood," was introduced by Sifneos^11^ to designate a cluster of cognitive and affective characteristics. It has been defined multidimensionally in terms of the following characteristics: difficulties in identifying and describing feelings, difficulties in discriminating between feelings and bodily sensations of emotional arousal, markedly constricted imaginative processes (as evidenced by a paucity of fantasies), a concrete and reality-based cognitive style (also denoted as externally oriented thinking), and a high degree of social conformity having little contact with one's own psychic reality. These characteristics have been hypothesized as being reflective of deficits in the mental representations of emotions and in the ability to regulate emotions through cognitive processes.^12^-^14^

To date, depression has been identified as the psychiatric disorder most consistently associated with alexithymia. Saarijärvi et al. revealed that alexithymia was significantly associated with depression,^15^ and that this association was relative stable over time,^16^,^17^ Honkalampi et al. found that depression might be the most important variable explaining the variance of alexithymia among general populations.^18^ In particular, depression was significantly associated with factor 2 (difficulty expressing feelings) of alexithymia.^17^,^19^ However, to our knowledge, few studies have focused on the association between alexithymia and stress response patterns among those with depressive disorders.

Therefore, this study was designed to evaluate how the presence of alexithymia, as measured by the Korean version of Toronto Alexithymia Scale-20 (TAS-20K),^20^ was related to their stress response patterns, as measured by the Stress Response Inventory (SRI),^9^ among individuals with depressive disorders. We hypothesized that alexithymia would result in poor emotional regulation and stress management abilities, thus leading poor or at least different stress response patterns among patients with depressive disorders.

## Methods

### Subjects

This study involved 98 patients diagnosed with depressive disorders. Of these, 51 were male and 47 were female, with a mean (±SD) age of 39.8 (±16.6) and a range of 18-75 years. The primary depressive diagnoses were major depressive disorder in 28 patients, dysthymic disorder in 49 patients, and depressive disorder not otherwise specified (NOS) in 21 patients. The mean (±SD) Clinical Global Impression (CGI)^21^ score was 3.4 (±0.9), indicating mild to moderate illness. Only nine patients were taking no psychiatric medications; 85 patients were taking therapeutic doses of antidepressants, most of whom (n=74) were also taking anxiolytic medications; three patients were taking only antipsychotic medications; and one patient was taking only anxiolytic medication. Table 1 shows the demographic and clinical characteristics of the sample.

### Procedures

All patients, who met the criteria of the Diagnostic and Statistical Manual of Mental Disorders-fourth edition (DSM-IV)^22^ for a primary diagnosis of depressive disorder, were evaluated in a single session with the Korean version of the 20-item Toronto Alexithymia Scale (TAS20K) and the SRI at Kyungpook National University Hospital, Daegu, Korea, between January 2003 and March 2007. At intake, diagnoses were ascertained using the Structured Clinical Interview for DSM-IV Axis I Disorders (SCID).^23^ Exclusion criteria included age under 18 years, mental retardation, and any neurological condition that might affect self-reporting.

Data on demographic and psychosocial factors (i.e., sex, age, and level of education), clinical profiles (i.e., primary and comorbid psychiatric diagnoses present during the evaluation according to DSM-IV criteria, duration of illness, medications and CGI scores), and results of psychological assessments (TAS-20K, SRI) were analyzed.

This study was approved by the institutional review board of Kyungpook National University Hospital.

### Psychological scales

#### The Korean Version of 20-Item Toronto Alexithymia Scale

The TAS-20 has become the most widely used tool for the measurement of alexithymia.^20^ This self-report questionnaire measures three intercorrelated dimensions of the alexithymia construct: difficulties identifying feelings, difficulties describing feelings, and externally oriented thinking. Each TAS-20 item was rated on a 5-point Likert scale, with total scores ranging from 20 to 100. The TAS-20 uses cutoff scoring: non-alexithymia, TAS score≦51; intermediate, 52≦TAS score≦60; and alexithymia, TAS score≧61.^14^ Note that the non-alexithymic group in this study included individuals who belonged to non-alexithymic as well as intermediate levels, that is, subjects with TAS score<61.

The study by Lee and colleagues^24^ reported how the TAS-20 was translated into Korean. Using confirmatory factor analysis, they showed that the three-factor structure of the original scale was consistent with the Korean version of the scale (Cronbach's α=0.76).

#### Stress Response Inventory

The SRI^9^ was developed by Koh et al. to measure multiple domains of stress responses, including emotional, somatic, cognitive, and behavioral arenas. The questionnaire contained 39 self-rated items, including seven subscales: tension, aggression, somatization, anger, depression, fatigue, and frustration. Each item was rated on a 5-point Likert scale. Test-retest reliability for scores on the seven subscales and for the total score was significantly high, ranging from 0.69 to 0.96. Cronbach's α for the seven subscales ranged from 0.76 to 0.91 and emerged as 0.97 for the total score.^9^

#### Clinical Global Impression-Severity Scale

The CGI-Severity (CGI-S)^21^ was used to assess the clinical impression of the current state of the patient's illness. The rater was asked to "consider his total clinical experience with the given population". The time span considered is the week prior to the rating; scores were recorded as follows: 1=normal, not at all ill, 2=borderline mentally ill, 3=mildly ill, 4=moderately ill, 5=markedly ill, 6=severely ill, and 7=among the most extremely ill patients.

### Statistical analyses

Between-group comparisons (alexithymia vs. non-alexithymia) were performed using chi-square analyses for categorical variables and independent t-tests for continuous variables. Because we conducted simultaneous tests for the seven subscales of the SRI, the significance level (p=0.05) was reduced to an α-adjusted p level of 0.007 (0.05/7) in all analyses of these subscales.

The scores of the seven subscales of the SRI, as well as factors such as gender, age, and duration of illness that emerged as significant in the between-group comparisons, were evaluated as potential covariates of categorical end-point (i.e., absence and presence of alexithymia) in a stepwise multivariate logistic regression with backward selection to identify the stress response factors associated with alexithymia. The backward selection model started with all candidate variables in the model. At each step, a variable that is not significant (p<0.10 by a likelihood ratio test) was removed. This process continued until no non-significant variables remained. Statistic analyses were performed using Statistical Package for the Social Science (SPSS) software (version 12.0; SPSS Inc., Chicago, IL). All significant levels were two-tailed and set at a 0.05 level.

## Results

### Between-group comparisons

Gender, age, and duration of illness showed significant differences between the alexithymia and non-alexithymia groups, whereas no significant differences between the two groups emerged in the level of education, primary Axis I diagnosis, presence of comorbid psychiatric disorder, and CGI score. Patients having depression with alexithymia scored significantly higher than those without alexithymia in all seven subscales of the SRI (Table 2).

### Logistic regression analysis

A logistic regression model was used to assess possible predictors for the presence of alexithymia in patients with depressive disorders. Factors included all seven subscales of the SRI, gender, age, and duration of illness. We found that aggression (B=0.25, Wald χ^2^=4.88, df=1, p=0.027) and somatization (B=0.31, Wald χ^2^=3.91, df=1, p=0.048), among the items in the SRI, as well as male sex (B=1.74, Wald χ^2^=5.46, df=1, p=0.019) were significantly associated with the presence of alexithymia in patients with depression. This model was 58% accurate in predicting the presence of alexithymia in those with depression (Table 3).

## Discussion

The main finding of this study was that patients with depression who had alexithymia were significantly different from those without alexithymia in terms of stress responses as well as in terms of clinical and demographic profiles. Patients having depression with alexithymia were more likely to be male and younger, qualify for a greater number of comorbid psychiatric disorders, and report shorter durations of illness than non-alexithymic counter-parts. The alexithymia group also showed more severe stress responses than the non-alexithymic group in all seven subscales of the SRI. More specifically, the presence of alexithymia was significantly associated with aggression, somatization, and male sex.

In general, alexithymic tendencies have been related to higher levels of anxiety, depression, and general psychological turmoil.^25^ As expected, our findings showed that patients with depression who had alexithymia obtained significantly higher scores than those without alexithymia in all seven subscales of the SRI. Although, to our knowledge, the SRI has not yet been used for direct comparisons between patients with depression, these findings are consistent with observations of Honkalampi et al.^26^ stating that patients having depression with alexithymia exhibited overall psychopathology (anxiety, hostility, psychotic symptoms, and somatization) and severe depression more frequently than their non-alexithymic counterparts. Note that no between-group differences were observed in CGI score and particular depressive diagnoses. In addition, Koh et al.^9^ used the SRI among a Korean sample and found that the group with depressive disorders was more susceptible to stress than any other groups, including those composed of individuals with anxiety, somatoform, and psychosomatic disorders. This greater susceptibility to stress among patients with depression may depend in part on the contributions of alexithymia.

The logistic regression analysis revealed that two subscales of SRI, somatization and aggression, in addition to male sex, were significantly associated with the presence of alexithymia in patients with depression. Individuals with alexithymia might be prone to functional somatic symptoms because of a tendency to amplify, focus on, and misinterpret the somatic sensations that accompany states of emotional arousal as well as other normal bodily sensations. Previous studies have suggested that individuals with alexithymia tended to develop dysphoric and functional somatic symptoms and were consequently more prone to compulsive affect-regulating behavior, such as binge-eating and psychoactive substance abuse.^27^-^29^ The latter would emerge as a coping mechanism in the context of difficulties with cognitively processing emotional and somatic stimuli. This study found that individuals with alexithymia showed more somatic stress responses even in the context of a sample confined to patients with depression.

Aggression, as measured by the SRI, was also associated with the presence of alexithymia. This subscale of the SRI consisted of four items (e.g., "I feel like hitting someone," "I feel like killing someone," "I feel like breaking something," and "I act violently as manifested, for example, in reckless driving, cursing, fighting").^9^ This finding indicated that alexithymia in patients with depression is directly associated with aggressive behaviors, and is congruent with previous observations that individuals with alexithymia experience chronic dysphoria or manifest outbursts of weeping or rage.^30^ Subjects with alexithymia may fail to recognize their feelings, and are more likely to use aggressive behaviors ineffectively. The "anger" subscale, however, was not significantly associated with the presence of alexithymia in this study. Sample items for this subscale included: "I hate someone," "I can't get that thought out of my mind," and "I get impatient easily". One possible explanation for this discrepancy may involve construing aggressive behaviors as overt manifestations of the dysregulation of emotions, particularly anger, conceptualized as a defining feature of alexithymia.^28^ Indeed, Koh et al. suggested that anger and aggression in particular were characteristic stress responses among those with depressive disorders.^31^ Our results elaborated on their findings insofar as they suggested that alexithymia-related difficulties in handling angry feelings would lead to aggressive behaviors in individuals with depression. One may infer that this finding might not reflect alexithymic tendencies per se, but rather the general behavioral tendencies among the demographic group consisting of young males. However, aggression remained significant even after adjusting for demographic variables in this study.

Our data also revealed that men who were depressed tended to be more alexithymic than women with depression. This finding was consistent with some studies,^32^-^35^ although other studies found no gender differences.^36^ A deficit in emotion-related language in men,^31^ or 'restrictive emotionality' deriving from the traditional socialized male gender role, has been proposed to explain this difference.^37^,^38^

This study is limited by the following. First, the cross-sectional design of this study, rendering causal inference impossible, represents a major limitation. Second, the sample consisted of patients at Kyungpook National University Hospital, rendering generalizations of the study results to the general psychiatric population problematic. More severe patients might have been overrepresented among those referred to a university hospital. Third, the association between alexithymic tendencies and stress response patterns was found among a sample composed of patients who were depressed. The depressive disorder group was not compared to groups of individuals diagnosed with other psychiatric disorders; thus, identifying alexithymic tendencies as the stress response pattern characterizing patients with depression in particular was impossible. Fourth, although the CGI score and the ratio of depressive disorder indirectly indicated two groups were comparable, a failure to control for the severity of depression and anxiety, which was significantly associated with alexithymia,^18^ complicated the interpretation of the results of group comparison.

Despite these limitations, our findings suggest that patients who have depression with alexithymia were more susceptible to stress than those without alexithymia. A regression analysis indicated that male sex, somatization, and aggression among patients who were depressed predicted alexithymic tendencies. Clinicians might improve the treatment of patients with depression by using these predictors of alexithymia to identify those who might benefit from developing and enhancing communication skills for coping with emotionally stressful situations.

## Figures and Tables

**TABLE 1:**
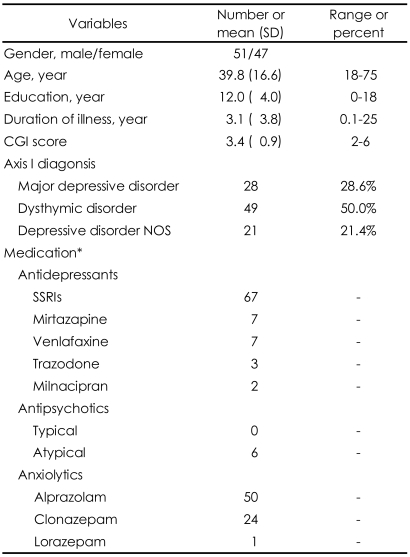
Demographic and clinical characteristics of patients with depressive disorder

^*^9 patients were not taking any psychiatric medications at the time of their psychological evaluation. CGI: Clinical Global Impression scale, NOS: not otherwise specified, SSRIs: selective serotonin reuptake inhibitors

**TABLE 2:**
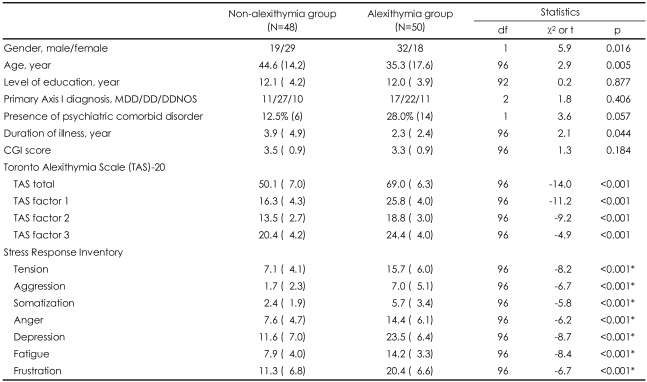
Comparison of non-alexithymia and alexithymia group among patients with depressive disorders

^*^Adjusted p value<0.05. MDD: major depressive disorder, DD: dysthymic disorder, DDNOS: depressive disorder NOS, CGI: clinical global impression, NOS: not otherwise specified

**TABLE 3:**
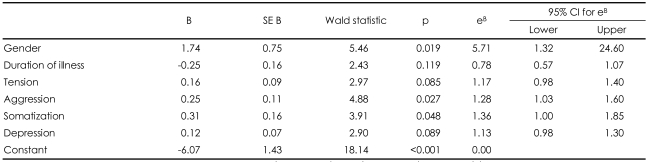
Logistic regression analysis predicting the presence of alexithymia

e^B^: exponentiated B, R^2^: 0.58 (Hosmer & Lemeshow), 0.55 (Cox & Snell), 0.74 (Negelkerke). Model χ^2^ (6)=78.54
